# The Genome Sequence of the Octocoral *Paramuricea clavata* – A Key Resource To Study the Impact of Climate Change in the Mediterranean

**DOI:** 10.1534/g3.120.401371

**Published:** 2020-07-13

**Authors:** Jean-Baptiste Ledoux, Fernando Cruz, Jèssica Gómez-Garrido, Regina Antoni, Julie Blanc, Daniel Gómez-Gras, Silvija Kipson, Paula López-Sendino, Agostinho Antunes, Cristina Linares, Marta Gut, Tyler Alioto, Joaquim Garrabou

**Affiliations:** * CIIMAR/CIMAR, Centro Interdisciplinar de Investigação Marinha e Ambiental, Universidade do Porto, Porto, 4050-123, Portugal; † Institut de Ciències del Mar, CSIC, Passeig Marítim de la Barceloneta 37-49, 08003 Barcelona, Spain; ‡ CNAG‐CRG, Centre for Genomic Regulation (CRG), The Barcelona Institute of Science and Technology, Baldiri Reixac 4, Barcelona, 08028, Spain; § University of Zagreb, Faculty of Science, Biology Department, Rooseveltov trg 6, 10000, Zagreb, Croatia; ** Department of Biology, Faculty of Sciences, University of Porto, Rua do Campo Alegre, 4169-007, Porto, Portugal; †† Departament de Biologia Evolutiva, Ecologia i Ciències Ambientals, Institut de Recerca de la Biodiversitat (IRBio), Universitat de Barcelona, Av. Diagonal 643, 08028 Barcelona, Spain; ‡‡ Universitat Pompeu Fabra (UPF), Doctor Aiguader 88, Barcelona, 08003, Spain; §§ Aix Marseille Univ, Université de Toulon, CNRS, IRD, MIO, Marseille, France

**Keywords:** *Paramuricea clavata*, octocoral, temperate habitat-forming anthozoan, mass mortality events, global warming, whole genome sequencing, *de novo* assembly, genome annotation, Oxford Nanopore Technologies, long read sequencing

## Abstract

The octocoral, *Paramuricea clavata*, is a habitat-forming anthozoan with a key ecological role in rocky benthic and biodiversity-rich communities in the Mediterranean and Eastern Atlantic. Shallow populations of *P. clavata* in the North-Western Mediterranean are severely affected by warming-induced mass mortality events (MMEs). These MMEs have differentially impacted individuals and populations of *P. clavata* (*i.e.*, varied levels of tissue necrosis and mortality rates) over thousands of kilometers of coastal areas. The eco-evolutionary processes, including genetic factors, contributing to these differential responses remain to be characterized. Here, we sequenced a *P. clavata* individual with short and long read technologies, producing 169.98 Gb of Illumina paired-end and 3.55 Gb of Oxford Nanopore Technologies (ONT) reads. We obtained a *de novo* genome assembly accounting for 607 Mb in 64,145 scaffolds. The contig and scaffold N50s are 19.15 Kb and 23.92 Kb, respectively. Despite of the low contiguity of the assembly, its gene completeness is relatively high, including 75.8% complete and 9.4% fragmented genes out of the 978 metazoan genes contained in the metazoa_odb9 database. A total of 62,652 protein-coding genes have been annotated. This assembly is one of the few octocoral genomes currently available. This is undoubtedly a valuable resource for characterizing the genetic bases of the differential responses to thermal stress and for the identification of thermo-resistant individuals and populations. Overall, having the genome of *P. clavata* will facilitate studies of various aspects of its evolutionary ecology and elaboration of effective conservation plans such as active restoration to overcome the threats of global change.

The red gorgonian, *Paramuricea clavata* (Risso 1826; Figure 1), is an octocoral belonging to the Holaxonia-Alcyoniina clade (McFadden *et al.* 2006) and thriving in the Mediterranean Sea and neighboring Atlantic Ocean from 15 to 200 m depth in dim light environment (Boavida *et al.* 2016). This species plays a key ecological role as a structural species in rocky-bottoms characterized by rich diverse Mediterranean coralligenous (Ballesteros 2006). Similar to trees in terrestrial forests, *P. clavata* generates three-dimensional structures that increase overall habitat complexity which in turn has a positive impact on associated species (Ponti *et al.* 2018). This long-lived species (up to 100 years) exhibits low population dynamics: it is characterized by recruitment by pulse, a slow growth rate (mean growth rate = 0.8 cm.years^-1^), late sexual maturity (10 years of age) (Linares *et al.* 2007) and restricted dispersal and re-colonization capacities (Mokhtar-Jamaï *et al.* 2011; Arizmendi-Mejía *et al.* 2015a). The red gorgonian populations are critically impacted by defaunation, due to habitat destruction (Linares *et al.* 2007), and warming-induced mass-mortality events (MMEs) (Garrabou *et al.* 2009). Considering the biology and ecology of the species, these pressures challenge the demographic and evolutionary responses of *P. clavata*. Consequently, *P. clavata* was recently included as a vulnerable species to the IUCN red list of Anthozoans in the Mediterranean (Otero *et al.* 2017). Moreover, there is a consensus among scientists and managers regarding the urgent need to develop new resources for this species in order to promote its conservation.

**Figure 1:**
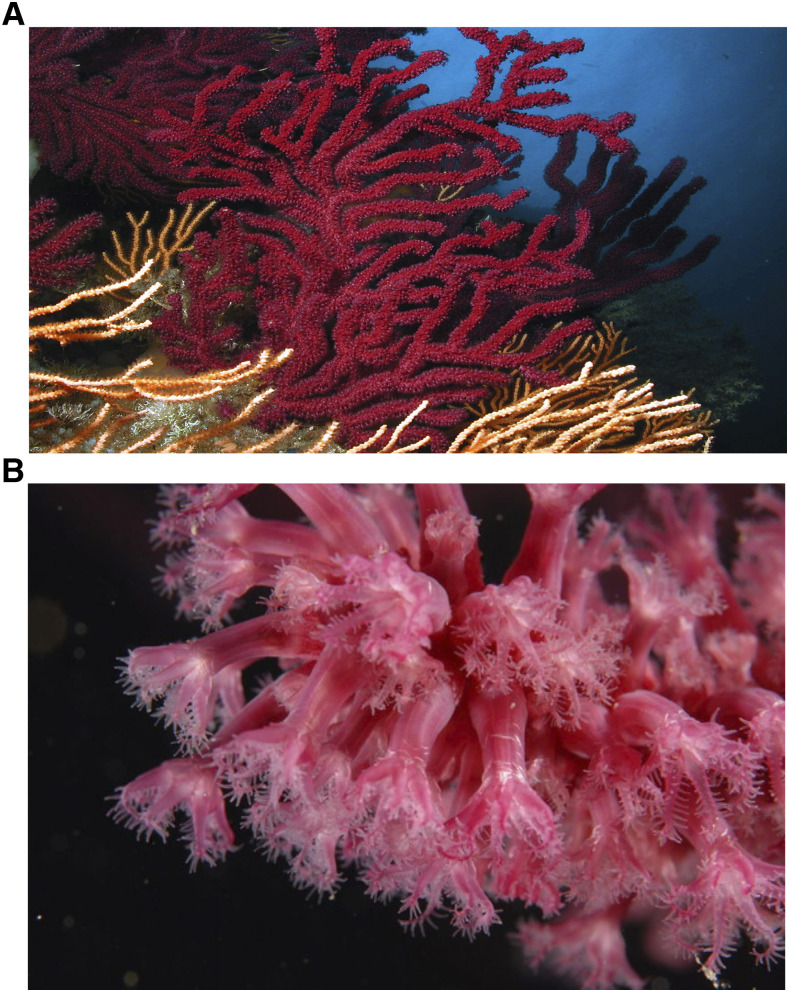
The red gorgonian *Paramuricea clavata* (Risso 1826): a) whole colony; b) close up on the polyps.

Focusing on MMEs, intensive field surveys have demonstrated the differential impact of warming on individuals and populations of *P. clavata*. For instance, during the 2003 MME, the percentage of affected colonies (*i.e.*, showing tissue necrosis) ranged from less than 5% up to more than 80% depending on the population (Garrabou *et al.* 2009). Thus, individuals and/or populations show different levels of tolerance to thermal stress, suggesting the occurrence of warming-resistant individuals. The presence of these individuals affords a new perspective for the conservation of the species, especially in terms of active restoration.

Accordingly, population-by-environment interactions (PEI) focused on the interactions with thermal environment have been receiving more attention in *P. clavata*. Common garden experiments in controlled conditions have been used to identify different physiological factors (*e.g.*, sex, sexual maturity) driving the differential responses to thermal stress reported from the field (Coma *et al.* 2009; Kipson 2013; Arizmendi-Mejía *et al.* 2015b). In the meantime, different studies have aimed to decipher the respective role of selected (local adaptation) and neutral (genetic drift) eco-evolutionary processes on the resistance to thermal stress (Ledoux *et al.* 2015; Crisci *et al.* 2017). However, definitive conclusions regarding the eco-evolution of thermo-resistance are still lacking mainly because of the limited genetic tools used (*e.g.*, low number of genetic markers). In order to promote the conservation of *P. clavata*, we aim to develop genomic resources to gain insights into the eco-evolutionary processes and genetic factors driving the differential response to thermal stress.

## Methods & Materials

### Sample collection

One apical tip (8 cm) of a reproductive colony (> 30 cm) of *P. clavata* was sampled by SCUBA diving at 20m depth in the Cova de la Vaca (42°2’52.97’’N; 3°13’34.76’’E) in The Montgrí, Medes Islands and Baix Ter Natural Park (Catalunya, Spain). The sample was transferred alive to the Experimental Aquaria Facility (ZAE) of the Institute of Marine Science (Barcelona; Spain) and placed in a 70L tank filled with filtered Mediterranean Sea water pumped from 10m depth in a continuous flux system. This sample was divided in three fragment sections (3_10-10, 5_27-10 and 6_2-1) for the DNA extractions described below.

### Genomic DNA extraction

Total genomic DNA was extracted from fresh tissue frozen in liquid nitrogen using the Gentra PureGene Tissue Kit (Qiagen) following manufacturer protocol. DNA purity and quantity were estimated using spectrophotometer and Qubit fluorescent based kit (Thermo Fisher Scientific). DNA integrity was assessed by 0.8% agarose gel electrophoresis.

### Whole genome sequencing with Illumina

The Roche-Kapa Biociences kit for short-insert paired-end (PE) libraries for Illumina was used for DNA library preparation of *P. clavata* with some minor modifications. A pool of seven gDNA extractions from fragment section 3_10-10 was re-purified with AMPure XP Beads (Agencourt, Beckman Coulter) and eluted in 50ul of water. Genomic DNA (6.0 μg) was sheared on a Covaris LE220 in order to reach DNA fragment sizes of ∼400-800bp. The fragmented DNA was size-selected on 1% agarose gel where eight bands were excised to isolate DNA fragments of precise insert size (520bp). Three gel fractions were selected for further purification with Qiagen QIAquick Gel Extraction Kit and the size was determined on an Agilent 2100 Bioanalyzer with the DNA7500 assay (362bp, 429bp, 548bp, fractions D, E, F), end-repaired, adenylated and ligated to dual matched indexed paired-end adaptors (IDT). The adaptor ligated library size (458bp, 516bp, 678bp) was confirmed on an Agilent 2100 Bioanalyzer with the DNA7500 assay. All libraries were quantified with the Library Quantification Kit for Illumina Platforms (Roche-Kapa Biosystems). The sequencing library with the mean insert size of 429bp (agarose gel, fraction E) was sequenced using TruSeq Rapid SBS Kit v2 (Illumina), in paired-end mode, 2x251bp, in two sequencing lanes of Illumina HiSeq2500 flowcell v2 (Illumina) according to standard Illumina operating procedures with a minimal yield of 170 Gb of raw data. Primary data analysis, image analysis, base calling and quality scoring of the run, were processed using the manufacturer’s software Real Time Analysis (RTA 1.18.66.3) and followed by generation of FASTQ sequence files by CASAVA.

### Long read whole genome sequencing

Genomic DNA of *P. clavata* was obtained from four extractions of fragment section 5_27-10 and six extractions from fragment section 6_2-1. All extractions from each respective fragment section were then pooled into two samples (pooled sample 5_27-10 and pooled sample 6_2-1) and re-purified using AMPure XP Beads (Agencourt, Beckman Coulter) adding 0.4 volume (V/V) to the pooled sample. Both pooled samples were quality controlled using Pippin Pulse (Sage Science) and with Nanodrop (Thermo Fisher Scientific) ratios 260/230 and 260/280. From each pooled sample a sequencing library was constructed using the Ligation Sequencing Kit 1D, SQK-LSK108 (Oxford Nanopore Technologies) starting with 2μg of restricted integrity gDNA without a fragmentation step. The DNA was repaired using the NEBNext FFPE Repair Mix (New England Biolabs), end-repaired and adenylated with the NEBNext Ultra II End Repair and A-Tailing Module (New England Biolabs) and MinION AMX adapters (Oxford Nanopore Technologies) were ligated using the NEB Blunt/TA Ligase Master Mix (New England Biolabs). Each step was followed by purification with AMPure XP Beads. The DNA/beads ratio was 1 (V/V) after the end-repair and adenylation step. After the repair and the final purification/size selection steps the DNA/beads ratio was 0.4 (V/V) in order to eliminate all fragments below 2kb.

The sequencing run was performed on a MinION instrument (Oxford Nanopore Technologies) using the R9.5 chemistry FLO-MIN107 flowcell for the first run (pooled sample 5_27-10) and the R9.4.1 chemistry FLO-MIN106 flowcell (Oxford Nanopore Technologies) for the second run (pooled sample 6_2-1), according to manufacturer’s recommendations. In brief, first the MinKNOW interface QC (Oxford Nanopore Technologies) was run in order to assess the flowcell quality and followed by flowcell priming. The sequencing library was mixed with running buffer, Library Loading Beads (Oxford Nanopore Technologies) and nuclease free water and loaded onto the “spot on” port for sequencing. The sequencing data were collected for 48 hr. The quality parameters of the sequencing runs were further monitored by the MinKNOW 1.10.16 platform while the run was base-called using the Albacore v2.0.1 agent in real time.

### Genome size and complexity

As there is no empirical estimate for *P. clavata* genome size, we downloaded 41 C-value estimates corresponding to the Cnidaria phylum in the Animal Genome Size Database (Gregory 2017). In addition, we ran two different k-mer analyses on the raw PE reads to estimate the ploidy (Supplementary Information), the size and the complexity of the genome. First, we examined the frequency distribution of 57-mers using Jellyfish v2.2.6 (Marçais and Kingsford 2011). Second, we determined the ploidy with Smudgeplot (Ranallo-Benavidez *et al.* 2020). Lastly, we ran GenomeScope v2.0 (Ranallo-Benavidez *et al.* 2020) using two different k-mer lengths (k = 21 and k = 57) and the appropriate ploidy level.

### Filtering contaminated reads and trimming Paired-End (PE) reads to 150 bp

Before *de novo* assembly, all reads from the paired-end library (PE400) were filtered of contaminants by mapping (gem-mapper (Marco-Sola *et al.* 2012) with up to 2% mismatches) against a contamination database that included phiX, Univec sequences, *E. coli* and various contaminants detected with Kraken (Wood and Salzberg 2014) in more than 0.01% of the reads (see Table 3). Note that our read decontamination method is stringent enough to remove real contaminants (almost exact matches with ≤ 2% mismatches) such as phiX but does not detect similar but divergent sequences (such as different bacterial strains); these are detected in the final assembly by using BLAST or during the genome annotation.

The filtered Illumina PE were trimmed to 150bp using FASTX toolkit v.0.0.13 (http://hannonlab.cshl.edu/fastx_toolkit/). This was done to optimize the *de Bruijn* graph construction. The trimming reduced the sequencing coverage to 144.42x (close to 120x – the ideal for a heterozygous genome) and increased the mean base quality of the reads (last cycles produce lower base qualities).

### De novo genome assembly

Two different hybrid assemblies were obtained with MaSuRCA v3.2.6 (Zimin *et al.* 2013, 2017): one using the complete PE400 2x251bp library (pcla1, Table S3) and a second using the reads trimmed to 150bp (pcla4, Table S3). In both cases, the reads were assembled with CELERA and the USE_LINKING_MATES option. As part of our genome annotation strategy (see below), we also removed 2,332 scaffolds contaminated with bacteria or fungi from pcla4 (our most contiguous hybrid assembly, see below). In addition, we mapped the complete mitochondrial genome (NC_034749.1) to the decontaminated assembly using minimap2 v2.14 (Li 2018), producing a complete mitochondrion (chrMT) and a nuclear genome assembly (pcla6, Table S3).

Finally, we collapsed the assembly with Purge Haplotigs v1.1.0 (Roach *et al.* 2018) in order to avoid the inclusion of redundant scaffolds corresponding to alternative *haplotigs*. The pre-processed PE400 reads were mapped against pcla6 with BWA MEM v.0.7.7 (Wood and Salzberg 2014) and the –M option to discard mappings of chimeric reads. Based on these mappings, we first plotted the coverage distribution with *purge_haplotigs hist* identifying three depth cut-offs: low = 32, midpoint = 143, high = 273 (Figure S4). Then scaffolds were flagged as ‘junk’ or ’suspect’ based on their coverage and the cut-offs used. The assembly was curated, discarding a total of 43,536 redundant scaffolds, and named pcla8 (Table S3).

### RNA library preparation and sequencing

To obtain a complete set of expressed genes for annotation, total RNA was extracted from three different individuals coming from three distant populations (LaVaca, Gargallu and Balun) from three regions (Catalan Sea, Corsica and Eastern Adriatic Sea) and submitted to a thermal stress in controlled conditions in aquaria. Sampling was conducted by scuba-diving between 20 and 35 m depth in each region. For each individual, an 8 cm apical tip was sampled (hereafter colony) and placed in coolers with seawater ice packs to maintain the water temperature between 15–18°. The colonies were transported alive to experimental aquaria facilities at the Institute of Marine Sciences-CSIC (Barcelona, Spain). The maximum transportation time was 36 h for the colonies collected in Gargallu (Corsica, France) and Balun (Eastern Adriatic, Croatia). Until the beginning of the experiment, colonies were maintained at control temperatures (16–17°) presenting expanded polyps during feeding and absence of tissue necrosis indicating their healthy condition. The experiment set-up was inspired by previous experiments with the same species (Arizmendi-Mejía *et al.* 2015b, Crisci *et al.* 2017). Briefly, after an acclimation period, the seawater was heated to 25° during 24h and maintained for 25 days. The tank was equipped with submersible pumps to facilitate water circulation. Temperature was registered with HOBO Water Temp v2 autonomous temperature sensors every half an hour. The experimental set functioned as an open system. Tissues were sampled and conserved in RNA later (Qiagen) for each individual before heating (T0) and after 25 days of thermal stress (T1). RNA extractions were conducted combining TRI Reagent Solution (Invitrogen) for tissue lysis and phase separation and Qiagen RNeasy Mini protocol for purification and elution. RNA extractions were pooled in two groups: one including the extractions at T0 and one including the extractions at T1 and quantified by Qubit RNA BR Assay kit (Thermo Fisher Scientific). RNA integrity was estimated by using the RNA 6000 Nano Bioanalyzer 2100 Assay (Agilent).

The RNASeq libraries were prepared from total RNA using KAPA Stranded mRNA-Seq Kit Illumina Platforms (Roche-Kapa Biosystems) with minor modifications. Briefly, after poly-A based mRNA enrichment with oligo-dT magnetic beads and 500ng of total RNA as the input material, the mRNA was fragmented. The strand specificity was achieved during the second strand synthesis performed in the presence of dUTP instead of dTTP. The blunt-ended double-stranded cDNA was 3′adenylated and Illumina platform-compatible adaptors with unique dual indexes and unique molecular identifiers (Integrated DNA Technologies) were ligated. The ligation product was enriched with 15 PCR cycles and the final library was validated on an Agilent 2100 Bioanalyzer with the DNA 7500 assay.

The libraries were sequenced on HiSeq 4000 (Illumina, Inc) with a read length of 2x76bp using HiSeq 4000 SBS kit in a fraction of a HiSeq 4000 PE Cluster kit sequencing flow cell lane generating a mean of 80 million paired end reads per sample. Image analysis, base calling and quality scoring of the run were processed using the manufacturer’s software Real Time Analysis (RTA 2.7.7).

### Genome annotation

First, repeats present in the pcla4 genome assembly were annotated with RepeatMasker v4-0-6 (http://repeatmasker.org) using the repeat library specific for our assembly that was built with RepeatModeler v1.0.11. Repeats that were part of repetitive protein families (detected by running a BLAST search of the repeat library against Swissprot) were removed from the library before masking the genome.

A first annotation of protein-coding genes was obtained by combining RNA-seq alignments, protein alignments and *ab initio* gene predictions on the pcla4 genome assembly. A flowchart of the annotation process is shown in Figure 5.

RNA-seq reads were aligned to the genome with STAR v-2.6.1b (Dobin *et al.* 2013) and transcript models were subsequently generated using Stringtie v1.0.4 (Pertea *et al.* 2015). PASA v2.3.3 (Haas *et al.* 2008) was used to combine the Stringtie transcript models with 534 soft coral nucleotide sequences downloaded from NCBI in January 2019. The *TransDecoder* program, which is part of the PASA package, was run on the PASA assemblies to detect coding regions in the transcripts. Then, the complete *Stylophora pistilata* proteome was downloaded from Uniprot (January 2019) and aligned to the genome using SPALN v2.3.1 (Gotoh 2008). *Ab initio* gene predictions were performed on the repeat-masked pcla4 assembly with four different programs: GeneID v1.4 (Parra *et al.* 2000), Augustus v3.2.3 (Stanke *et al.* 2006), GlimmerHMM (Majoros *et al.* 2004) and Genemark-ES v2.3e (Lomsadze *et al.* 2014) with and without incorporating evidence from the RNA-seq data. All the gene predictors except Genemark, which runs in a self-trained manner, were run with the parameters obtained by training with a set of high-quality candidate genes extracted from the Transdecoder results. Finally, all the data were combined into consensus CDS models using EvidenceModeler-1.1.1 (Haas *et al.* 2008). Additionally, UTRs and alternative splicing forms were annotated through two rounds of PASA annotation updates. The resulting transcripts were clustered into genes using shared splice sites or significant sequence overlap as criteria for designation as the same gene. Functional annotation of the annotated proteins was done using Blast2go (Conesa *et al.* 2005), which in turn ran a BLASTP (Altschul *et al.* 1997) search against the non-redundant database (March 2019) and Interproscan (Jones *et al.* 2014) to detect protein domains on the annotated proteins.

Finally, the annotation of non-coding RNAs (ncRNAs) was performed as follows. First, the program cmsearch (v1.1) (Cui *et al.* 2016) which is part of the Infernal package (Nawrocki and Eddy 2013) was run against the RFAM (Nawrocki *et al.* 2015) database of RNA families (v12.0). Also, tRNAscan-SE (v1.23) (Lowe and Eddy 1997) was run in order to detect the transfer RNA genes present in the genome assembly. PASA-assemblies longer than 200bp that had not been annotated as protein-coding and not overlapped by more than 80% by a small ncRNA were incorporated into the ncRNA annotation as long-non-coding RNAs (lncRNAs).

Via the functional annotation, we were able to detect the presence of bacterial and fungal genes, suggesting the presence of some residual contamination in the assembly (pcla4). Therefore, we removed potential contaminant sequence by combining as criteria for retention the gene functional annotation, the mean GC content and the presence of expression and *P. clavata*-specific repeats for each scaffold. As a result of this decontamination process, 2,322 scaffolds were removed from the assembly as they belonged mainly to *Aspergillus*, *Endozoicomonas* or other bacteria (pcla5, Table S3). Finally, after separating the mitochondrial genome and collapsing haplotypes with Purge Haplotigs, the annotation of the resulting assembly (pcla8) was updated by removing the genes present in the discarded scaffolds.

The gene completeness of the pcla8 assembly (*i.e.*, free of contaminants, mitochondrion and redundant haplotigs) was estimated with BUSCO v3 (Simão *et al.* 2015) using the metazoa database (metazoa_odb9) of 978 conserved genes.

### Orthofinder

In order to understand the origin of the larger-than-expected number of annotated genes, we ran Orthofinder (Emms and Kelly 2019) to determine Orthogroups and Orthologs between all pcla8 genes and those of *Renilla muelleri* (Jiang *et al.* 2019) and *Stylophora pistillata* (Voolstra *et al.* 2017). Finally, the GO enrichment for the genes not classified in orthogroups was estimated with topGO (Alexa *et al.* 2006).

### Genome-wide heterozygosity (SNVs)

Re-sequencing at sufficient depth (>20x) allows the extraction of valuable genome-wide information from a single diploid sample by simply re-mapping against the reference genome and calling variants. Although adaptor removal and quality trimming are not recommended for MaSuRCA, they are strictly necessary before variant calling. Therefore, we detected and trimmed Illumina adaptor sequences and performed quality trimming of the PE400 library. For this purpose, we used the Trim Galore! wrapper script (http://www.bioinformatics.babraham.ac.uk/projects/trim_galore/) with -q 10 and then we filtered out contaminated reads, as described above (Table 3). Finally, all these reads were mapped against a reference genome that included pcla8 and chrMT using BWA MEM with option –M.

For variant calling, we used GATK 3.7 (McKenna *et al.* 2010; DePristo *et al.* 2011), adapting the “GATK Best Practices HaplotypeCaller GVCF” (Van der Auwera *et al.* 2013) to a diploid organism without a set of known variants such as the Single Nucleotide Polymorphism database (dbSNP). Specifically, we did not perform base quality score recalibration, as the model normally degrades the base qualities in the absence of known variant sites. First, the BWA alignment was screened for duplicates using MarkDuplicates of PICARD v1.6 (https://broadinstitute.github.io/picard/). Then, we identified the callable sites per sample using the GATK’s CallableLoci tool, with options–minBaseQuality 10–minMappingQuality 20, to be in concordance with the default value for these parameters in HaplotypeCaller (see below). The actual variant calling was performed using the HaplotypeCaller but restricting it to callable sites and with options: -dt NONE -rf BadCigar–never_trim_vcf_format_field -ploidy 2–min_base_quality_score 10–standard_min_confidence_threshold_for_calling 30–emitRefConfidence GVCF and–GVCFGQBands at Genotype Qualities 15, 20, 25, 30, 35, 40, 45, 50, 55, 60, 65, 70, 80 and 99. The resulting GVCF was used to call genotypes with the GenotypeGVCF tool and option–never_trim_vcf_format_field.

After variant calling, we exclusively considered supported SNVs, defined as those that are bi-allelic, covered at least by 10 reads and at least two reads supporting the alternative allele at heterozygous sites. The k-mer analyses (see Figure S3b) shows that a small amount of artificially duplicated sequences (k-mers) remain in the assembly, in both the homozygous and the heterozygous peaks. These duplicated sites may contribute to an underestimation of heterozygosity because the reads corresponding to each allele can potentially map separately to two independent locations in the assembly rather than to the same locus. For this reason, we extracted all 57-mers repeated exactly twice in the heterozygous and homozygous peaks of the assembly (pcla8). Our approach used first JELLYFISH v2.2.6 to dump all 57-mers contained in the PE reads (with coverage between 20x and 200x, Figure S3) into a FASTA file. Second, we ran KAT v2.3.3 (Mapleson *et al.* 2017) (kat sect with options -F -G 2) to get all 57-mers contained in the FASTA repeated exactly twice. The third step consisted in obtaining the location of these artificially duplicated 57-mers by looking for exact mappings with GEM-mapper build 1.81 (parameters -e 0 -m 0 -s 0). Afterward, these genomic intervals were merged into a BED file using BEDTools v.2.16.2 (Quinlan and Hall 2010) and subtracted from the callable sites with BEDOPS/2.0.0a (Neph *et al.* 2012). Finally, we selected all SNV variants at callable sites that did not contain artificially duplicated 57-mers using GATK v.3.7 to estimate the *corrected* SNV heterozygosity rate.

### Microsatellite markers

Previous molecular markers were identified and used for the species without information about their exact location in the genome. The newly sequenced and annotated genome has provided the means to do this. In order to know the genomic context around these markers, we mapped the 18 available microsatellite markers (Mokhtar-Jamaï *et al.* 2011; Ledoux unpublished data) to pcla8 using our BLAST server (http://denovo.cnag.cat/genomes/pclavata/blast/).

### Data availability

The assembly and raw reads have been deposited in the European Nucleotide Archive under the project accession PRJEB33489. The assembly, annotation, genome browser and BLAST server are also accessible via denovo.cnag.cat/pclavata. Supplemental material available at figshare: https://doi.org/10.25387/g3.12640691.

## Results and Discussion

### Whole genome sequencing with Illumina and Nanopore sequencing technologies

The Illumina HiSeq2500 run produced 338.6 million pairs of 251bp reads, accounting for a total of 169.98 Gb of sequence, representing 242.76x coverage of the genome (Table 1). The total nanopore yield accounted for 3.55 Gb (5x coverage) and read lengths were relatively low, with a read N50 of 2.67 Kb. It is noteworthy that the efficiency of the nanopore sequencing was heavily influenced by the relatively low quality of the DNA extraction (Figure S1).

**Table 1:** Whole genome sequencing output

Library	Read length (bp)	Fragment length (bp)	Yield (Gb)	Coverage*^a^*	error r1 (%)	error r2 (%)
PE400	251	395	169.98	242.76	0.29	0.46
Oxford Nanopore (1D reads)*^b^*	2,677	—	3.55	5.07	16.12	—

a Sequencing coverage has been estimated assuming the largest genome size estimate: 700.18 Mb.

b Information corresponding to the 1D reads produced by two MinION runs. The read N50 is 2,677 and N90 is 1,253 bp. The error rate for Illumina estimated from differences with respect to phix control, while the ONT error rate is estimated from differences with respect to lambda phage control sequence.

### Genome size and complexity

**Figure 2:**
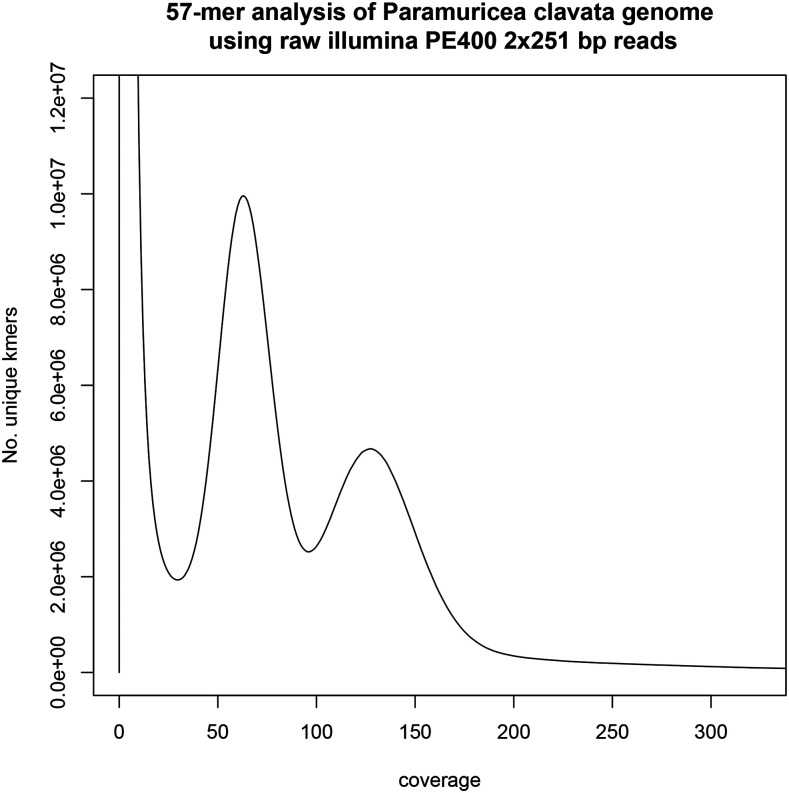
57-mer analysis of the sequenced genome. All 57-mers in the PE400 library were counted and the number of distinct 57-mers (kmer species) for each depth from 1 to 250 are shown in this plot. The main homozygous peak at depth 124 corresponds to unique homozygous sequence and a tall heterozygous peak lies at half depth of it (62). Finally, the thick long tail starting at depth 180 corresponds with repetitive k-mers in the genome. The high peak at very low depths, caused by sequencing errors, has been truncated to facilitate representation.

**Figure 3:**
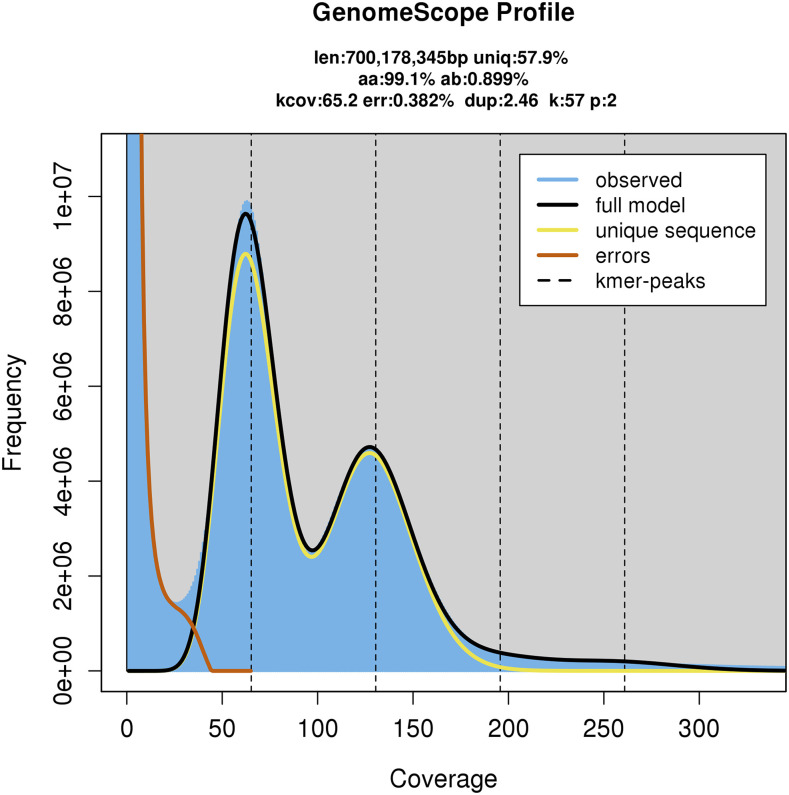
k-mer profile and model fit as estimated with GenomeScope v.2.0 from PE400 using a k-mer length of 57 bp. Note that the model finds an excess of repetitive sequence in the rightmost tail of the distribution after depth 180.

We show the results of two GenomeScope analyses and compare them with the empirical data available for the phylum Cnidaria. GenomeScope fits the 21-mer and the 57-mer profiles to a diploid mixture model (Model Fit: 52.23–94.81% and 66.57-96.50, respectively). This method estimates the haploid genome size to be between 610.08 and 700.17 Mb (Table 2). The analysis also suggests that the genome possess an appreciable amount of heterozygosity (0.89–1.59%) and high repetitiveness (42.09–56.42% of the genome is likely to be repeated) (Figures 2, 3 and S3b).

**Table 2:** Genome properties

	k = 21		k = 57	
Genome Property*^a^*	min	max	min	max
Homozygous (%)	98.40	98.46	99.09	99.11
Heterozygous (%)	1.53	1.59	0.89	0.91
Genome Haploid Length (bp)	610,087,141	612,695,236	697,960,926	700,178,345
Genome Repeat Length (bp)	344,214,541	345,686,043	293,794,366	294,727,750
Genome Unique Length (bp)	265,872,600	267,009,193	404,166,560	405,450,595
Model Fit (%)	52.23	94.81	66.57	96.50
Read Error Rate (%)	0.61	0.61	0.38	0.38
Genome with Repeats (%)	56.42	56.42	42.09	42.09

a Estimated from the Illumina pre-processed PE400 2x251bp reads by GenomeScope v.2.0 using a diploid model (*P* = 2) with k = 21 and k = 57, respectively.

The genome size of cnidarians shows large variations, ranging from just 224.92 Mb in *Nematostella vectensis* (T.R. Gregory, unpublished data) to the 3.48 Gb estimated for *Agalma elegans* (Adachi *et al.* 2017). However, the mean cnidarian genome size is 838.45 Mb, and the closest estimate to *P. clavata* belongs to *Sarcophyton sp*. (another Antozoan member of the Order Alcyonacea), which has a haploid genome size of 625.94 Mb (Adachi *et al.* 2017) suggesting the value of 610.08-612.69 Mb is likely a reliable estimate of *P. clavata* genome size.

### De novo genome assembly

The most contiguous assembly was obtained using 144.42x coverage with the 2x150 bp trimmed reads and 5x of ONT long-read data (*i.e.*, pcla4). We interpret this in two different but related ways. First, trimming 100bp off of the reads results in a reduction of the number of error k-mers that complicate the construction of the *de Bruijn* graph. Second, *de Bruijn* assemblers work best up to 50-80x coverage [probably even 100x (*e.g.*, Desai *et al.* 2013) or 120x for heterozygous genomes; Vurture *et al.* 2017], above which spurious contigs begin to appear due to the presence of more sequencing errors [*e.g.*, Desai *et al.* 2013; Mirebrahim *et al.* 2015; Lonardi *et al.* 2015]. Therefore, trimming and coverage reduction have jointly contributed to obtain a much cleaner *de Bruijn* graph, and subsequently better super-reads to be aligned to the long reads. In fact, the contiguity of the super-reads built by MaSuRCA for pcla1 is lower than for pcla4. These super-reads have N50 538bp and 573bp, respectively.

**Figure 4:**
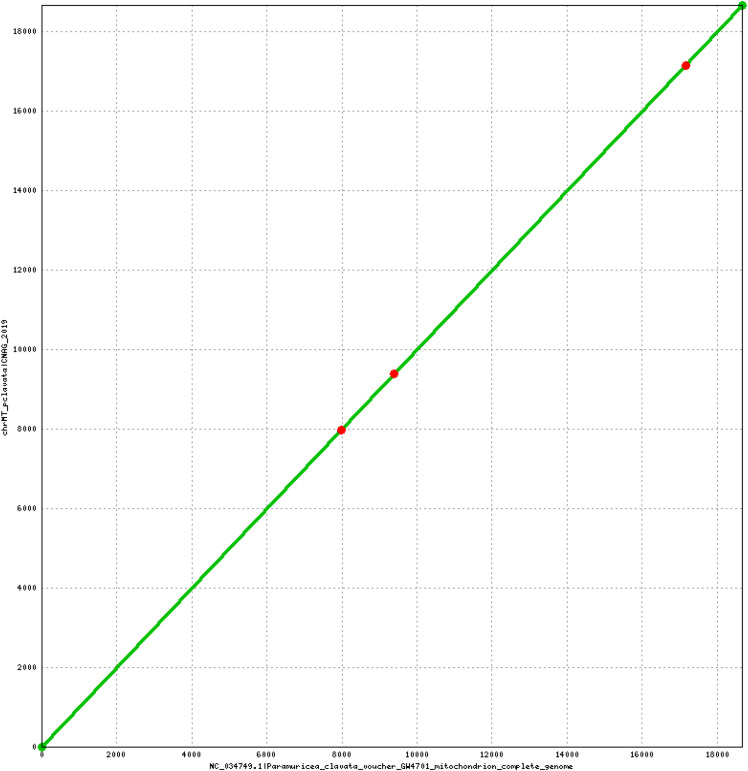
Alignment of the complete mitochondrial assembly against the NCBI reference genome (NC_034749.1) using DNAdiff v1.2 (MUMMER 3.22 package (Kurtz *et al.* 2004)). The figure produced with Mummerplot v3.5 (MUMMER 3.22 package) shows the location of the three mismatches found: one indel at position 9,389 plus two SNVs at positions 7,977 and 17,155, respectively.

The selected hybrid assembly (pcla4) comprises 724.62 Mb and has contig N50 (ctgN50) 15.86 Kb and scaffold N50 (scfN50) 19.72 Kb. We found only one scaffold aligning to the mitochondrial reference with two large mitochondrial segments repeated. This scaffold was removed from the assembly and the alternative mitochondrial genome (chrMT) was kept separately. Our mitochondrial assembly matches the reference genome with 99.8% identity. In fact, the few differences are restricted to one indel and two Single Nucleotide Variants (SNVs) (Figure 4).

**Figure 5:**
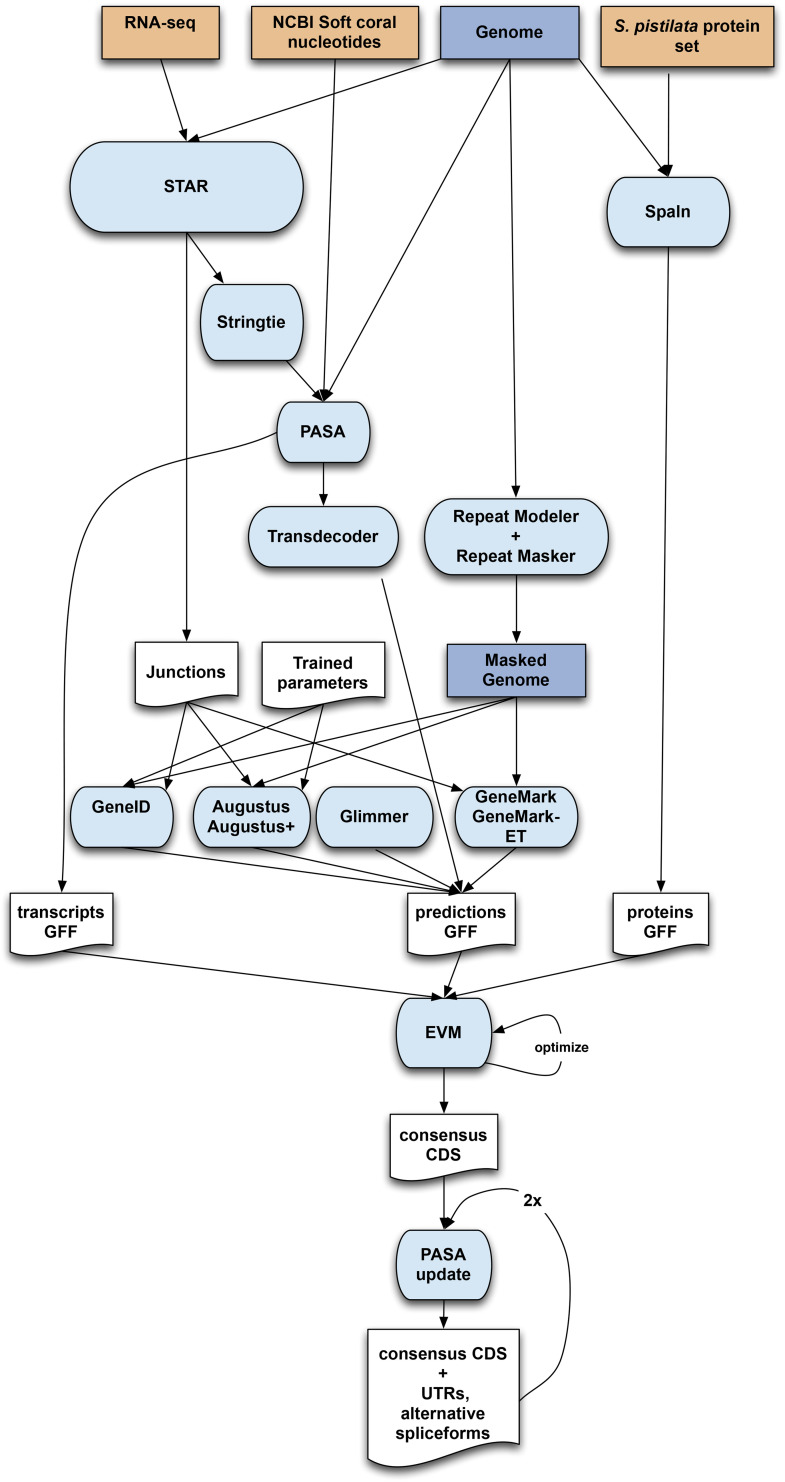
Genome annotation workflow.

After decontamination and removal of haplotigs (pcla8), the total assembly length was reduced to 606.96 Mb, very close to the Genomescope2 estimate using a diploid model (619.69 Mb) and the *Sarcophyton sp*. haploid genome length (625.9 Mb). Complete statistics are shown in Table 4. However, a closer inspection of the spectra copy number (KAT; Marçais and Kingsford 2011), suggests low levels of artificial duplications due to uncollapsed haplotypes (violet tip on top of the homozygous peak; Figure S3b). At the same time, though, it appears that true repeats have not been collapsed (bi-color tail evidencing the inclusion of 2x and 3x repeats; Figure S3b), likely due to the use of long read data. Consistent with the KAT results, the mapping rate of the Illumina PE400 against pcla6 is also very high, accounting for 97.76% of the total reads mapping to it.

In summary, the final assembly (pcla8) appears to be quite complete in terms of sequence and has a total length that is very similar to that estimated by the 21-mer analysis and the *Sarcophyton* sp. C-value (Table 3, 4). However, it is still highly fragmented with 64,145 scaffolds and scaffold N50 close to 24 Kb. The reasons for this are likely the short length (N50 = 2.67Kb) and the sub-optimal coverage of nanopore reads: less than 10x (Zimin *et al.* 2017).

**Table 3:** Sequence contaminants

Species	% reads covered	No. reads covered*^a^*	No. reads assigned	NCBI TaxID
*Alteromonas mediterranea**^b^*	7.86	393,091	393,091	314275
*Methanobacterium formicicum*	0.47	23,547	23,547	2162
*Candida dubliniensis*	0.36	17,802	0	42374
*Leishmania major*	0.35	17,613	0	5664
*Salmonella enterica*	0.16	7,871	0	28901
*Myceliophthora thermophila*	0.15	7,378	0	78579
*Streptococcus sp. VT 162*	0.14	6,921	6,921	1419814
*Saccharomyces cerevisiae*	0.1	5,153	0	4932
*Theileria orientalis*	0.07	3,293	0	68886
*Thielavia terrestris*	0.06	2,824	0	35720
*Kluyveromyces lactis*	0.04	2,018	2,018	28985
*Human herpesvirus 7*	0.03	1,475	1,475	10372
*Cyprinid herpesvirus 1*	0.03	1,282	1,282	317858
*Mycobacterium kansasii*	0.02	902	0	1768
*Methanococcus voltae*	0.02	787	0	2188
*Tadarida brasiliensis circovirus 1*	0.02	783	783	1732201

a The total number of read pairs screened was 5 million.

b Actually included 10 matches to *A. mediterranea* (Taxonomy ID: 314275) and 393,081 matches to *A. mediterranea U8* (Taxonomy ID: 1300257). Therefore, 393,081 matches represent 7.86% of the total reads.

**Table 4:** Contiguity of the assembly (pcla8)

	Contigs		Scaffolds	
	Length (bp)	Number	Length (bp)	Number
**N0**	205,335	1	239,170	1
**N5**	81,130	290	96,122	248
**N10**	61,509	728	73,949	613
**N15**	50,628	1,276	61,101	1,069
**N20**	42,871	1,928	51,904	1,609
**N25**	36,585	2,695	45,142	2,238
**N30**	31,872	3,584	39,216	2,958
**N35**	27,922	4,602	34,575	3,785
**N40**	24,634	5,760	30,608	4,718
**N45**	51,732	7,072	27,035	5,774
**N50**	19,152	8,558	23,918	6,967
**N55**	16,816	10,247	21,053	8,322
**N60**	14,652	12,176	18,511	9,861
**N65**	12,776	14,393	16,101	11,618
**N70**	10,959	16,956	13,818	13,654
**N75**	9,226	19,970	11,705	16,037
**N80**	7,541	23,599	9,613	18,893
**N85**	5,898	28,129	7,593	22,437
**N90**	4,201	34,184	5,486	27,108
**N95**	2,396	43,517	3,181	34,239
**N100**	128	79,709	203	64,145
**Total**	606,183,174	79,709	606,969,498	64,145

### Genome annotation

A total of 49% of the genome assembly was annotated as repetitive. Table 5 shows the proportions of each repeat type for the pcla8 assembly (notice that they sum to more than 49% as some positions fall in more than one category). The BUSCO analyses for gene completeness reports 75.8% complete genes (73.4% single-copy and 2.4% duplicated), 9.4% fragmented genes and 14.8% missing genes. In total, the *P. clavata* genome assembly contains 62,652 annotated protein-coding genes, which produce 70,788 transcripts (1.13 transcripts per gene) and encode for 69,836 unique protein products. The annotated transcripts have 4.92 exons on average, with 65.1% of them being multi-exonic (Table 6). In addition, 46,784 non-coding transcripts have been annotated, of which 23,443 and 23,431 are long and short non-coding RNA genes, respectively.

**Table 5:** Repeat annotation

Repeat type	Bases covered	% genome
LINE	33,645,094	5.54
SINE	9,570,518	1.58
DNA	65,436,874	10.78
LTR	8,780,644	1.45
RC	1,374,121	0.23
Satellite	1,422,244	0.23
SnRNA	86,345	0.01
Simple repeat	1,303,146	0.21
Unknown	196,068,308	32.30

**Table 6:** Genome annotation statistics

Number of protein-coding genes	62,652
Median gene length (bp)	1,634
Number of transcripts	70,788
Number of exons	288,028
Number of coding exons	277,455
Coding GC content	41.58%
Median UTR length (bp)	571
Median intron length (bp)	382
Exons/transcript	4.92
Transcripts/gene	1.13
Multi-exonic transcripts	65.1%
Gene density (gene/Mb)	103.22

The high number of protein coding genes compared to other octocoral species (*e.g.*, around 23,000 genes in *Renilla muelleri*; Jiang *et al.* 2019) may have several non-exclusive explanations, including: real or artificial genome duplication, mis-annotation of transposable elements, contamination or assembly fragmentation. In order to shed light on this issue, we defined orthogroups and orthologs among our annotation and those of *Renilla muelleri* (Octocorallia; 23,660 genes) and *Stylophora pistillata* (Hexacorallia; 24,067 genes). Orthofinder results report 12,419 orthogroups, 12,061 (97.1%) of which contain at least one *P. clavata* gene (see Fig S5a), supporting the high completeness of our genome. Moreover, the total number of *P. clavata* genes assigned to orthogroups (23,611; see Fig S5b) is closer to the values reported to date in Anthozoans.

The main factor contributing to the high number of apparent orphan genes (39,041) is likely the low contiguity of the assembly, with only minor contributions from duplication, contamination and mis-annotation of transposable elements. Duplicated genes should in principle be placed in orthogroups. Regarding contamination, it is noteworthy that orphan genes are distributed throughout the assembly, in many cases sharing scaffolds with genes that have been placed in orthogroups. A GO enrichment of these genes shows several enriched categories related mainly to chromosomal and telomere organization, meiosis and immune response but not to transposable element categories (see Table S4).

As for fragmentation, 34% of the annotated genes contain partial Open-Reading frames (ORFs) and 35% are mono-exonic suggesting that at least 35% of the annotated genes are fragmented. Comparing the orphan and the partial genes, we demonstrated a 66% overlap of the two categories. This suggests that around 2/3 of orphan genes result from the high fragmentation of the genome and supports the number of genes assigned to orthogroups (23,611) as a relevant estimate of the number of protein-coding genes in *P. clavata*.

### Genome-wide heterozygosity (SNVs) and microsatellite markers

Our corrected estimate of the heterozygosity rate is 0.71% and is defined as the total number of heterozygous SNVs (3,052,169) divided by the total number of callable sites that do not contain artificially duplicated 57-mers (429,483,187 bp). Thus, on average, we expect to find approximately 7 SNVs per Kb (or 7,160 per Mb) in this species, an estimate that is close to the 0.9% obtained with GenomeScope2 with k = 57 (Table 2).

With the exception of Pcla-26, all the microsatellite markers previously identified in *P. clavata* have been correctly assembled and located in our genome (Table S5). The repeat unit of Pcla-20 seems to be (TA) instead of (TTAT). Pcla-09, -14, -20, -24, -25 apart, twelve microsatellites were associated to protein sequences. While Pcla-a, -10, -12, -17, -20, -22, -28, and -29 were found entirely within intronic sequences, either one or both primers or the repeat sequence of Pcla-d, Pc3-81, Pcla -21, Pcla-23 and Pcla-27 fall within exonic sequences (see Table S5).

## Conclusion

The genomic and transcriptomic resources developed here open up new ways to study on the ecology and evolution of *Paramuricea clavata* and related octocoral species. In particular, we aim to characterize the eco-evolutionary processes, including the genomic factors, involved in the differential responses to thermal stress observed during the warming-induced mass-mortality events. We are currently re-sequencing the genome of thermo-resistant and sensitive individuals identified through a transregional common garden experiment developed in the framework of the MERCES project (European Union’s Horizon 2020 research and innovation program http://www.merces-project.eu). The targeted individuals belong to 12 populations from five distant regions in the Mediterranean, Adriatic and Eastern Atlantic. This work will be complemented by gene expression analyses at the transcriptomic level involving some of the re-sequenced individuals. In parallel, we are developing a holobiont approach and the genomic resources will complement microbiome analyses to reveal the temporal and spatial interactions between *P. clavata* and its associated micro-eukaryote and prokaryote symbionts (*e.g.*, La Rivière *et al.* 2013). We also aim to refine our existing knowledge on the evolutionary and demographic history of the species and are currently expanding our sampling to regions that were not covered by the common garden experiment (*e.g.*, Turkey, Algeria, South West of Spain). In the end, the genome assembly described here will directly enable applications such as active restoration or assisted evolution (van Oppen *et al.* 2015) aimed at the conservation of *P. clavata*. Moreover, considering the structural role of *P. clavata* in biodiversity-rich coralligenous communities, future results obtained using these genomic and transcriptomic resources should extend to and benefit these communities as well.

## References

[bib1] Adachi K., Miyake H., Kuramochi T., Mizusawa K., and Okumura S., 2017 Genome size distribution in phylum Cnidaria. Fish. Sci. 83: 107–112. 10.1007/s12562-016-1050-4

[bib2] Alexa A., Rahnenfuhrer J., and Lengauer T., 2006 Improved scoring of functional groups from gene expression data by decorrelating GO graph structure. Bioinformatics 22: 1600–1607. 10.1093/bioinformatics/btl140 16606683

[bib3] Altschul S. F., Madden T. L., Schäffer A. A., Zhang J., Zhang Z., 1997 Gapped BLAST and PSI-BLAST: A new generation of protein database search programs. Nucleic Acids Res. 25: 3389–3402. 10.1093/nar/25.17.3389 9254694 PMC146917

[bib4] Arizmendi-Mejía R., Linares C., Garrabou J., Antunes A., Ballesteros E., 2015 a Combining genetic and demographic data for the conservation of a mediterranean marine habitat-forming species. PLoS One 10: e0119585 10.1371/journal.pone.0119585 25774522 PMC4361678

[bib5] Arizmendi-Mejía R., Ledoux J.-B., Civit S., Antunes A., Thanopoulou Z., 2015 b Demographic responses to warming: reproductive maturity and sex influence vulnerability in an octocoral. Coral Reefs 34: 1207–1216. 10.1007/s00338-015-1332-9

[bib6] Van der Auwera G. A., Carneiro M. O., Hartl C., Poplin R., del Angel G., 2013 From FastQ Data to High-Confidence Variant Calls: The Genome Analysis Toolkit Best Practices Pipeline, pp. 11.10.1–11.10.33 in Current Protocols in Bioinformatics, John Wiley & Sons, Inc, Hoboken, NJ. 10.1002/0471250953.bi1110s43 PMC4243306 25431634

[bib7] Ballesteros E., 2006 Mediterranean coralligenous assemblages: A synthesis of present knowledge. Oceanogr. Mar. Biol. 44: 123–195. 10.1201/9781420006391.ch4

[bib8] Boavida J., Assis J., Silva I., and Serrão E. A., 2016 Overlooked habitat of a vulnerable gorgonian revealed in the Mediterranean and Eastern Atlantic by ecological niche modelling. Sci. Rep. 6: 36460 10.1038/srep36460 27841263 PMC5107895

[bib9] Coma R., Ribes M., Serrano E., Jiménez E., Salat J., 2009 Global warming-enhanced stratification and mass mortality events in the Mediterranean. Proc. Natl. Acad. Sci. USA 106: 6176–6181. 10.1073/pnas.0805801106 19332777 PMC2669359

[bib10] Conesa A., Gotz S., Garcia-Gomez J. M., Terol J., Talon M., 2005 Blast2GO: a universal tool for annotation, visualization and analysis in functional genomics research. Bioinformatics 21: 3674–3676. 10.1093/bioinformatics/bti610 16081474

[bib11] Crisci C., Ledoux J.-B., Mokhtar-Jamaï K., Bally M., Bensoussan N., 2017 Regional and local environmental conditions do not shape the response to warming of a marine habitat-forming species. Sci. Rep. 7: 5069 10.1038/s41598-017-05220-4 28698582 PMC5505982

[bib12] Cui X., Lu Z., Wang S., Jing-Yan Wang J., and Gao X., 2016 CMsearch: simultaneous exploration of protein sequence space and structure space improves not only protein homology detection but also protein structure prediction. Bioinformatics 32: i332–i340. 10.1093/bioinformatics/btw271 27307635 PMC4908355

[bib13] DePristo M., Banks E., Poplin R., Garimella K. V., Maguire J. R., 2011 A framework for variation discovery and genotyping using next-generation DNA sequencing data. Nat. Genet. 43: 491–498. 10.1038/ng.806 21478889 PMC3083463

[bib14] Desai A., Marwah V. S., Yadav A., Jha V., Dhaygude K., 2013 Identification of Optimum Sequencing Depth Especially for De Novo Genome Assembly of Small Genomes Using Next Generation Sequencing Data. PLoS One 8: e60204 10.1371/journal.pone.0060204 23593174 PMC3625192

[bib15] Dobin A., Davis C. A., Schlesinger F., Drenkow J., Zaleski C., 2013 STAR: ultrafast universal RNA-seq aligner. Bioinformatics 29: 15–21. 10.1093/bioinformatics/bts635 23104886 PMC3530905

[bib16] Emms D. M., and Kelly S., 2019 OrthoFinder: phylogenetic orthology inference for comparative genomics. Genome Biol. 20: 238 10.1186/s13059-019-1832-y 31727128 PMC6857279

[bib17] Garrabou J., Coma R., Bensoussan N., Bally M., Chevaldonné P., 2009 Mass mortality in Northwestern Mediterranean rocky benthic communities: Effects of the 2003 heat wave. Glob. Change Biol. 15: 1090–1103. 10.1111/j.1365-2486.2008.01823.x

[bib18] Gotoh O., 2008 A space-efficient and accurate method for mapping and aligning cDNA sequences onto genomic sequence. Nucleic Acids Res. 36: 2630–2638. 10.1093/nar/gkn105 18344523 PMC2377433

[bib19] Gregory T., 2020 Animal Genome Size Database. http://www.genomesize.com

[bib20] Haas B. J., Salzberg S. L., Zhu W., Pertea M., Allen J. E., 2008 Automated eukaryotic gene structure annotation using EVidenceModeler and the Program to Assemble Spliced Alignments. Genome Biol. 9: R7 10.1186/gb-2008-9-1-r7 18190707 PMC2395244

[bib21] Jiang J. B., Quattrini A. M., Francis W. R., Ryan J. F., Rodríguez E., 2019 A hybrid de novo assembly of the sea pansy (Renilla muelleri) genome. Gigascience 8: 1–7. 10.1093/gigascience/giz026 PMC6446218 30942866

[bib22] Jones P., Binns D., Chang H.-Y., Fraser M., Li W., 2014 InterProScan 5: genome-scale protein function classification. Bioinformatics 30: 1236–1240. 10.1093/bioinformatics/btu031 24451626 PMC3998142

[bib23] Kipson, S., 2013 Ecology of gorgonian dominated communities in the Eastern Adriatic Sea. PhD thesis, University of Zagreb, Zagreb, Croatia. 160p.

[bib24] Kurtz S., Phillippy A., Delcher A. L., Smoot M., Shumway M., 2004 Versatile and open software for comparing large genomes. Genome Biol. 5: R12 10.1186/gb-2004-5-2-r12 14759262 PMC395750

[bib25] La Rivière M., Roumagnac M., Garrabou J., and Bally M., 2013 Transient shifts in bacterial communities associated with the temperate gorgonian *Paramuricea clavata* in the Northwestern Mediterranean Sea. PLoS One 8: e57385 10.1371/journal.pone.0057385 23437379 PMC3577713

[bib26] Ledoux J.-B., Aurelle D., Bensoussan N., Marschal C., Féral J.-P., 2015 Potential for adaptive evolution at species range margins: Contrasting interactions between red coral populations and their environment in a changing ocean. Ecol. Evol. 5: 1178–1192. 10.1002/ece3.1324 25859324 PMC4377262

[bib27] Li H., 2018 Minimap2: pairwise alignment for nucleotide sequences. Bioinformatics 34: 3094–3100 10.1093/bioinformatics/bty191 29750242 PMC6137996

[bib28] Linares C., Doak D. F., Coma R., Díaz D., and Zabala M., 2007 Life history and viability of long-lived marine invertebrate: the octocoral *Paramuricea clavata*. Ecology 88: 918–928 10.1890/05-1931 17536708

[bib29] Lomsadze A., Burns P. D., and Borodovsky M., 2014 Integration of mapped RNA-Seq reads into automatic training of eukaryotic gene finding algorithm. Nucleic Acids Res. 42: e119 10.1093/nar/gku557 24990371 PMC4150757

[bib30] Lonardi S., Mirebrahim H., Wanamaker S., Alpert M., Ciardo G., 2015 When less is more: ‘slicing’ sequencing data improves read decoding accuracy and *de novo* assembly quality. Bioinformatics 31: 2972–2980. 10.1093/bioinformatics/btv311 25995232

[bib31] Lowe T. M., and Eddy S. R., 1997 tRNAscan-SE: A Program for Improved Detection of Transfer RNA Genes in Genomic Sequence. Nucleic Acids Res. 25: 955–964. 10.1093/nar/25.5.955 9023104 PMC146525

[bib32] Majoros W. H., Pertea M., and Salzberg S. L., 2004 TigrScan and GlimmerHMM: two open source ab initio eukaryotic gene-finders. Bioinformatics 20: 2878–2879. 10.1093/bioinformatics/bth315 15145805

[bib33] Mapleson D., Garcia Accinelli G., Kettleborough G., Wright J., and Clavijo B. J., 2017 KAT: a K-mer analysis toolkit to quality control NGS datasets and genome assemblies. Bioinformatics 33: 574–576. 10.1093/bioinformatics/btw663 27797770 PMC5408915

[bib34] Marçais G., and Kingsford C., 2011 A fast, lock-free approach for efficient parallel counting of occurrences of k-mers. Bioinformatics 27: 764–770. 10.1093/bioinformatics/btr011 21217122 PMC3051319

[bib35] Marco-Sola S., Sammeth M., Guigó R., and Ribeca P., 2012 The GEM mapper: fast, accurate and versatile alignment by filtration. Nat. Methods 9: 1185–1188. 10.1038/nmeth.2221 23103880

[bib36] McFadden C. S., France S. C., Sánchez J. A., and Alderslade P., 2006 A molecular phylogenetic analysis of the Octocorallia (Cnidaria: Anthozoa) based on mitochondrial protein-coding sequences. Mol. Phylogenet. Evol. 41: 513–527. 10.1016/j.ympev.2006.06.010 16876445

[bib37] McKenna A., Hanna M., Banks E., Sivachenko A., Cibulskis K., 2010 The Genome Analysis Toolkit: A MapReduce framework for analyzing next-generation DNA sequencing data. Genome Res. 20: 1297–1303. 10.1101/gr.107524.110 20644199 PMC2928508

[bib38] Mirebrahim H., Close T. J., and Lonardi S., 2015 *De novo* meta-assembly of ultra-deep sequencing data. Bioinformatics 31: i9–i16. 10.1093/bioinformatics/btv226 26072514 PMC4765875

[bib39] Mokhtar-Jamaï K., Pascual M., Ledoux J.-B., Coma R., Féral J.-P., 2011 From global to local genetic structuring in the red gorgonian Paramuricea clavata: the interplay between oceanographic conditions and limited larval dispersal. Mol. Ecol. 20: 3291–3305. 10.1111/j.1365-294X.2011.05176.x 21762434

[bib40] Nawrocki E. P., and Eddy S. R., 2013 Infernal 1.1: 100-fold faster RNA homology searches. Bioinformatics 29: 2933–2935. 10.1093/bioinformatics/btt509 24008419 PMC3810854

[bib41] Nawrocki E. P., Burge S. W., Bateman A., Daub J., Eberhardt R. Y., 2015 Rfam 12.0: updates to the RNA families database. Nucleic Acids Res. 43: D130–D137. 10.1093/nar/gku1063 25392425 PMC4383904

[bib42] Neph S., Kuehn M. S., Reynolds A. P., Haugen E., Thurman R. E., 2012 BEDOPS: high-performance genomic feature operations. Bioinformatics 28: 1919–1920. 10.1093/bioinformatics/bts277 22576172 PMC3389768

[bib43] van Oppen M. J. H., Oliver J. K., Putnam H. M., and Gates R. D., 2015 Building coral reef resilience through assisted evolution. Proc. Natl. Acad. Sci. USA 112: 2307–2313. 10.1073/pnas.1422301112 25646461 PMC4345611

[bib44] Otero M. M., Numa C., Bo M., Orejas C., Garrabou J., 2017 Overview of the conservation status of Mediterranean anthozoans, Malaga, Spain. x + 73.

[bib45] Parra G., Blanco E., and Guigó R., 2000 GeneID in Drosophila. Genome Res. 10: 511–515. 10.1101/gr.10.4.511 10779490 PMC310871

[bib46] Pertea M., Pertea G. M., Antonescu C. M., Chang T.-C., Mendell J. T., 2015 StringTie enables improved reconstruction of a transcriptome from RNA-seq reads. Nat. Biotechnol. 33: 290–295. 10.1038/nbt.3122 25690850 PMC4643835

[bib47] Ponti M., Turicchia E., Ferro F., Cerrano C., and Abbiati M., 2018 The understorey of gorgonian forests in mesophotic temperate reefs. Aquat. Conserv. 28: 1153–1166. 10.1002/aqc.2928

[bib48] Quinlan A. R., and Hall I. M., 2010 BEDTools: a flexible suite of utilities for comparing genomic features. Bioinformatics 26: 841–842. 10.1093/bioinformatics/btq033 20110278 PMC2832824

[bib49] Ranallo-Benavidez T. R., Jaron K. S., and Schatz M. C., 2020 GenomeScope 2.0 and Smudgeplot for reference-free profiling of polyploid genomes. Nat. Commun. 11: 1432 10.1038/s41467-020-14998-3 32188846 PMC7080791

[bib50] Roach M. J., Schmidt S. A., and Borneman A. R., 2018 Purge Haplotigs: allelic contig reassignment for third-gen diploid genome assemblies. BMC Bioinformatics 19: 460 10.1186/s12859-018-2485-7 30497373 PMC6267036

[bib51] Voolstra C. R., Li Y., Liew Y. J., Baumgarten S., Zoccola D., 2017 Comparative analysis of the genomes of Stylophora pistillata and Acropora digitifera provides evidence for extensive differences between species of corals. Sci. Rep. 7: 17583 10.1038/s41598-017-17484-x 29242500 PMC5730576

[bib52] Simão F. A., Waterhouse R. M., Ioannidis P., Kriventseva E. V., and Zdobnov E. M., 2015 BUSCO: assessing genome assembly and annotation completeness with single-copy orthologs. Bioinformatics 31: 3210–3212. 10.1093/bioinformatics/btv351 26059717

[bib53] Simpson J. T., 2014 Exploring genome characteristics and sequence quality without a reference. Bioinformatics 30: 1228–1235. 10.1093/bioinformatics/btu023 24443382 PMC3998141

[bib54] Stanke M., Schöffmann O., Morgenstern B., and Waack S., 2006 Gene prediction in eukaryotes with a generalized hidden Markov model that uses hints from external sources. BMC Bioinformatics 7: 62 10.1186/1471-2105-7-62 16469098 PMC1409804

[bib55] Vurture G. W., Sedlazeck F. J., Nattestad M., Underwood C. J., Fang H., 2017 GenomeScope: fast reference-free genome profiling from short reads. Bioinformatics 33: 2202–2204. 10.1093/bioinformatics/btx153 28369201 PMC5870704

[bib56] Wood D. E., and Salzberg S. L., 2014 Kraken: ultrafast metagenomic sequence classification using exact alignments. Genome Biol. 15: R46 10.1186/gb-2014-15-3-r46 24580807 PMC4053813

[bib57] Zimin A. V., Marçais G., Puiu D., Roberts M., Salzberg S. L., 2013 The MaSuRCA genome assembler. Bioinformatics 29: 2669–2677. 10.1093/bioinformatics/btt476 23990416 PMC3799473

[bib58] Zimin A. V., Puiu D., Luo M.-C., Zhu T., Koren S., 2017 Hybrid assembly of the large and highly repetitive genome of Aegilops tauschii, a progenitor of bread wheat, with the MaSuRCA mega-reads algorithm. Genome Res. 27: 787–792. 10.1101/gr.213405.116 28130360 PMC5411773

